# HER2 Targeted Molecular MR Imaging Using a *De Novo* Designed Protein Contrast Agent

**DOI:** 10.1371/journal.pone.0018103

**Published:** 2011-03-24

**Authors:** Jingjuan Qiao, Shunyi Li, Lixia Wei, Jie Jiang, Robert Long, Hui Mao, Ling Wei, Liya Wang, Hua Yang, Hans E. Grossniklaus, Zhi-Ren Liu, Jenny J. Yang

**Affiliations:** 1 Department of Chemistry, Georgia State University, Atlanta, Georgia, United States of America; 2 Department of Biology, Georgia State University, Atlanta, Georgia, United States of America; 3 Department of Radiology, Emory University, Atlanta, Georgia, United States of America; 4 Department of Ophthalmology, Emory University School of Medicine, Atlanta, Georgia, United States of America; Medical College of Georgia, United States of America

## Abstract

The application of magnetic resonance imaging (MRI) to non-invasively assess disease biomarkers has been hampered by the lack of desired contrast agents with high relaxivity, targeting capability, and optimized pharmacokinetics. We have developed a novel MR imaging probe targeting to HER2, a biomarker for various cancer types and a drug target for anti-cancer therapies. This multimodal HER20targeted MR imaging probe integrates a *de novo* designed protein contrast agent with a high affinity HER2 affibody and a near IR fluorescent dye. Our probe can differentially monitor tumors with different expression levels of HER2 in both human cell lines and xenograft mice models. In addition to its 100-fold higher dose efficiency compared to clinically approved non-targeting contrast agent DTPA, our developed agent also exhibits advantages in crossing the endothelial boundary, tissue distribution, and tumor tissue retention over reported contrast agents as demonstrated by even distribution of the imaging probe across the entire tumor mass. This contrast agent will provide a powerful tool for quantitative assessment of molecular markers, and improved resolution for diagnosis, prognosis and drug discovery.

## Introduction

Molecular imaging specifically probes the molecular abnormalities of diseases to allow earlier detection, monitoring of disease progression, and molecular assessment of treatments [1]. Molecular imaging using the modality of magnetic resonance imaging (MRI) has significant advantages in pre-clinical research and clinical diagnosis and prognosis as MRI offers superior spatial resolution without depth limitation, exquisite soft tissue contrast, clinical availability, while avoiding ionizing radiation [2]. However, many applications of MRI rely on the administration of contrast agents to amplify the contrast of the interested regions to obtain both sensitivity and specificity [3]. Developing contrast agents that can be specifically targeted to various biomarkers allowing real-time imaging of biological events at the molecular level will have great clinical importance [4], [5], [6]. To achieve molecular imaging by MRI, especially to quantitatively monitor the expression level of the disease biomarkers, it is essential to develop contrast agents with high relaxivity, target capability, optimized pharmacokinetics, tissue penetration and low or no toxicity [7].

Human epidermal growth factor receptor (EGFR) type 2 (HER2/neu) is a cell surface receptor of the EGF family that is overexpressed in breast, ovarian, urinary bladder and many other carcinomas. In the case of breast cancer, HER2 overexpression is typically associated with younger patients and generally poor prognoses with substantially higher probabilities of relapse after treatment [8], [9]. In addition, the HER2 mediated recognition system has been widely employed as a drug target for anti-cancer therapies. Unfortunately, current diagnosis of HER-2 positive tumor relies mostly on the use of fine needle biopsies with subsequent immunohistochemistry (IHC) analysis and/or fluorescent in situ hybridization (FISH). These methods suffer from several drawbacks including sampling errors, misinterpretation due to lack of quantization, and discordance between primary tumors and metastases. Thus, assessment of HER2/neu levels by non-invasive MR imaging will provide a tremendous tool for cancer diagnosis/prognosis, design of treatment strategies, and monitoring the effectiveness of the treatment.

## Results

### Metal binding affinity and relaxivity

**Figure 1:**
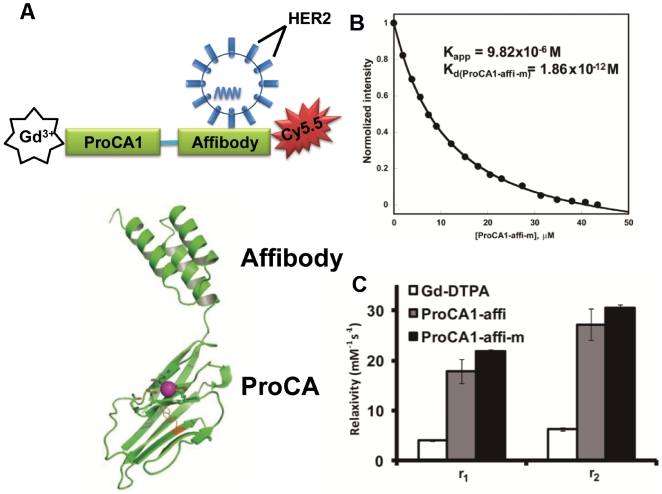
Design and properties of multimodality HER2-targeted protein contrast agent ProCA1-affi for *in vivo* cancer targeting and imaging by MRI and NIR. (**A**) The model structure of multimodal HER2 targeted MR imaging probe created by connecting a high affinity HER2 affibody Z_HER2-342_ at the C-terminal of a *de novo* designed protein contrast agent ProCA1.CD2 with a designed Gd^3+^ binding site. A near IR fluorescence dye Cy5.5 was then conjugated to the added Cys at the C-terminal of the fusion protein. The designed probe was further modified by a tri-branched polyethylene glycol (PEG) with 40 PEG subunits (ProCA1-affi-m). (**B**) The metal binding affinity for Gd^3+^ with K_d_ of 1.86×10^−12^ M was measured by competitive method using Fluo5N [10] (**C**) The relaxivity of ProCA1-affi with (gray) and without PEGylation (ProCA1-affi-m, black) and clinically used Gd-DTPA (white) were measured under the magnetic field of 1.41 T at 37°C (*P<0.05). The developed contrast agent exhibited 5–6 fold greater relaxivity in both r_1_ and r_2_. *P is the value from Student's t-test.

We have developed a novel multimodal molecular imaging probe to target cancer marker HER2/neu using magnetic resonance and near infrared imaging (Fig. 1). We employed a protein-based MRI contrast moiety (ProCA1) that was developed by de novo designing the Gd3+ binding site(s) into a stable host protein, the domain 1 of rat CD2 (10 KDa). Due to the unique features of the designed metal binding properties, the protein contrast agent exhibited a significant improved T1 relaxivity for MRI contrast enhancement compared to that of commonly used Gd-DTPA (Diethylenetriamine Pentaacetic Acid) at 1.4–4.7T field strength [10]. A high affinity HER2 affibody [11], [12] was engineered into the C terminal of the designed Gd3+-binding protein by a flexible linker. The small molecular size (16 KDa) provides good tissue penetration. We also introduced an optical imaging capability by conjugating a near-IR dye Cy5.5 to a Cys residue at C-terminal of the protein to facilitate imaging analyses (Fig. 1A). To increase protein solubility, blood circulation time, and reduction of immunogenicity, the designed HER2 targeting protein contrast agent was PEGylated using PEG-40, a molecule with tri-branches of 12 units PEG (denoted as ProCA1-affi-m, Fig. 1A).

The designed MRI contrast agent was expressed in *E. coli* and subsequently purified (Supporting File S1). Similar to the parental protein ProCA1.CD2, the designed protein (ProCA1-affi) had a strong metal binding affinity with Kd for Gd^3+^ at 1.87×10–12 M [10] (Fig. 1B). ProCA1-affi also exhibited r1 and r2 relaxivities of 21 and 30 mM^−1^s^−1^ at 1.41 T, respectively (Fig. 1B). The developed protein with conjugated NIR dye exhibited fluorescence excitation and emission maxima at 640 and 695 nm, respectively, and excitation coefficient constant of 0.21 µM^−1^cm^−1^ (Supporting File S1). Far UV CD and fluorescent spectra analyses indicated that the developed contrast agent is well folded (Supporting File S1). The toxicity of the designed protein was analyzed with CD-1 mice. No acute toxicity was observed following tail vein injections of 4-fold greater dosages than that currently used in MRI, evaluated over a 2-day test period. Characterization of serum samples from the test mice receiving the agent detected no apparent damage to kidney, liver, or heart (Supporting File S1).

### Cancer cell targeting capability

**Figure 2:**
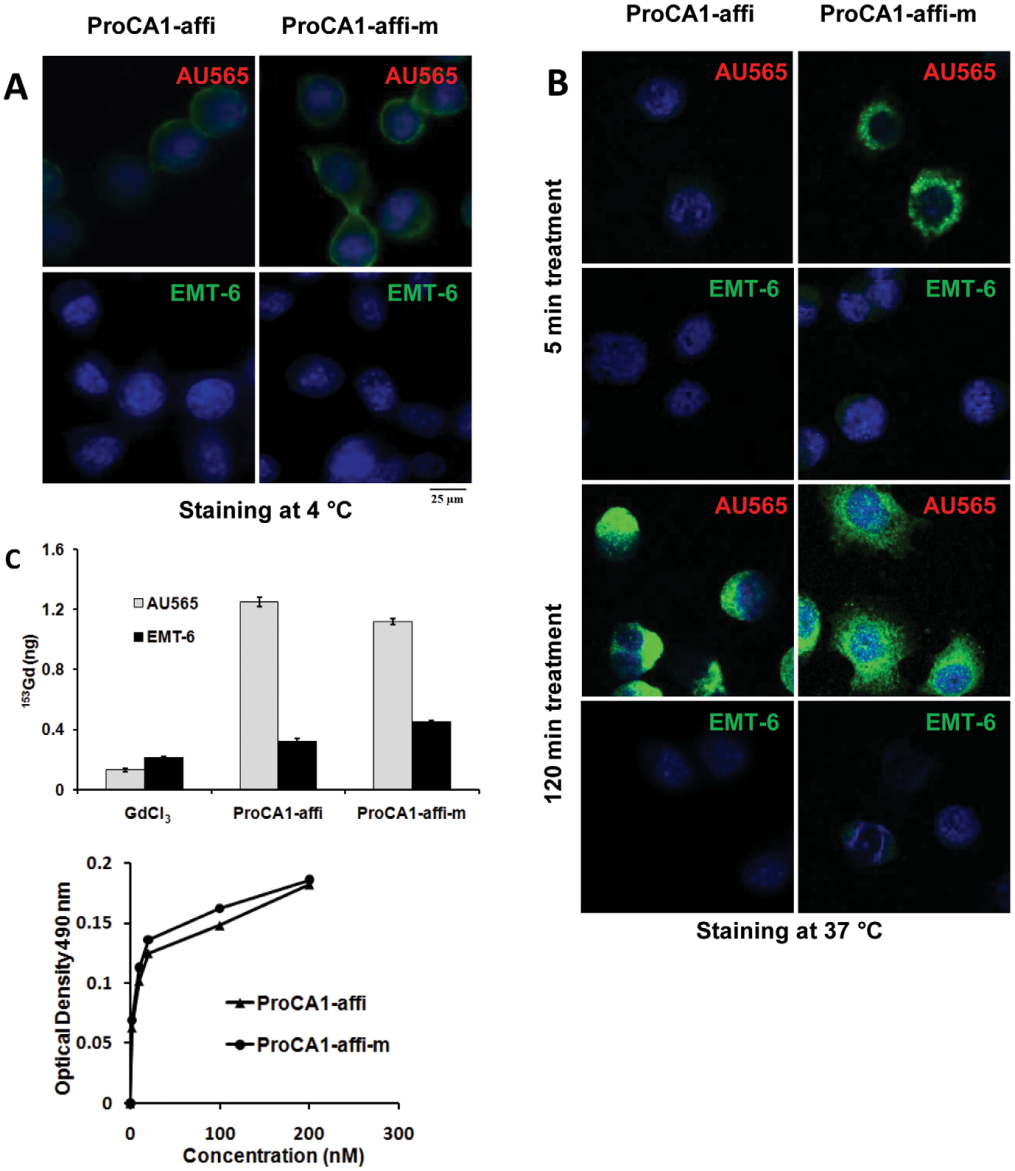
Examination of targeting capability in cultured cancer cells. (**A**) At 4°C, the HER2 positive (AU565) and negative (EMT-6) cancer cells were treated with ProCA1- affi and ProCA1 -affi-m respectively for 2 hours. The HER2 expressed on the cell membrane of AU565 was revealed by the green color from the goat-anti-rabbit secondary antibody (Invitrogen) for self-generated rabbit antibody against ProCA1-affi-m. The blue color shows the nuclear staining. (**B**). At 37°C, the cancer cells with HER2 positive (AU565) and negative (EMT-6) were treated with ProCA1 -affi and ProCA1 -affi-m respectively for 5 min and 2 hours. The immunefluorescence staining studies revealed that ProCA1- affi and ProCA1- affi-m bind to HER2 positive cell extensively and were largely relocated into the cytosol via endocytosis after 2 hours (green color). The blue color shows the nuclear staining. At both 4 and 37°C, negative staining was obtained in EMT-6 cells that lack HER2 expression. (**C**) (top) ^153^GdCl_3_, and^153^Gd loaded ProCA1 -affi and ProCA1-affi-m were incubated with cultured cancer cells for 2 hours. After careful washing, the radioactive signaling in the cell pellets was measured using γ-counter. The retention of ProCA1-CD2-affi or ProCA1-CD2-affi-m with Gd^3+^ in HER2 positive cells (AU565) was ∼3–4 folds greater than that in the HER2 negative cells (EMT-6) and non-specific uptake in ^153^GdCl_3_ treated cells. (bottom) ELISA assay revealed that the specific binding of HER2 positive cells by the developed contrast agents enhanced upon increasing the contrast agent concentrations.

We next examined whether the designed ProCA1-affi can target to cancer cells by cell binding analyses. We used three human cancer cell lines, AU565, SKOV-3 and MDA-MB-231. AU565 is a human breast cancer cell line, with HER2 expression level 1×10^6^ HER2/cell. SKOV-3 is an ovarian cancer cell line with estimated 3×10^6^ HER2/cell [13]. MBD-MDA-231 is a breast cancer cell line with modest HER2 levels (∼3×10^4^ HER2/cell) [14]. EMT-6 is a HER2 negative mouse breast cancer cell line. Binding of the Gd-ProCA1-affi to the selected cells was first analyzed by immuno-fluorescence staining using the polyclonal antibody against PEGylated parental protein ProCA1 (PAbPGCA1) (Fig. 2). A substantial staining intensity of ProCA1-affi bound to AU565 cells was observed and increased as incubation times increased. In contrast, the EMT-6 cells demonstrated very weak staining (Fig. 2A). It was evident that the Gd^3+^ ProCA1-affi bound to the cell surface HER2 with a clear membrane staining pattern in AU565 cells at 4°C (Fig. 2A). However, binding of the Gd^3+^ proteins to the cells triggered receptor mediated internalization at 37°C as demonstrated by the staining of the intracellular ProCA1-affi. The majority of the contrast agents entered the cells after 120 minutes incubation (Fig. 2B). The Gd^3+^ ProCA1-affi was stable after internalization at 120 minutes, indicating that the designed Gd^3+^ ProCA1-affi withstood protein degradation during and after endocytosis. The immunostaining results were consistent with NIR fluorescence imaging results (Supporting File S1). Binding of the Gd^3+^ ProCA1-affi to the two testing cell lines were further analyzed by quantification of cell bound Gd^3+^ by γ-counting the trace of isotope ^153^Gd^3+^- in the Gd^3+^ ProCA1-affi complexes (Fig. 2C). The results supported our immuno-analyses that Gd^3+^ ProCA1-affi was retained 3-4 folds greater in HER2 positive AU565 cells than HER2 negative EMT-6 cells (Fig. 2). Measuring the amount of bound Gd^3+^ from γ-counting revealed that the Gd^3+^ ions were bound to cells at ∼0.1 fmole Gd/cell (P<0.01). Under assumption that 1×10^7^ cells make a volume of 50–100 µl, this binding capacity led to the accumulation of Gd^3+^ at 10–20 µM in the cell pellets. This local concentration is sufficient to produce strong MRI contrast, especially with the high relaxivity protein contrast agent reported here.

### *In vivo* imaging on xenograft mice

**Figure 3:**
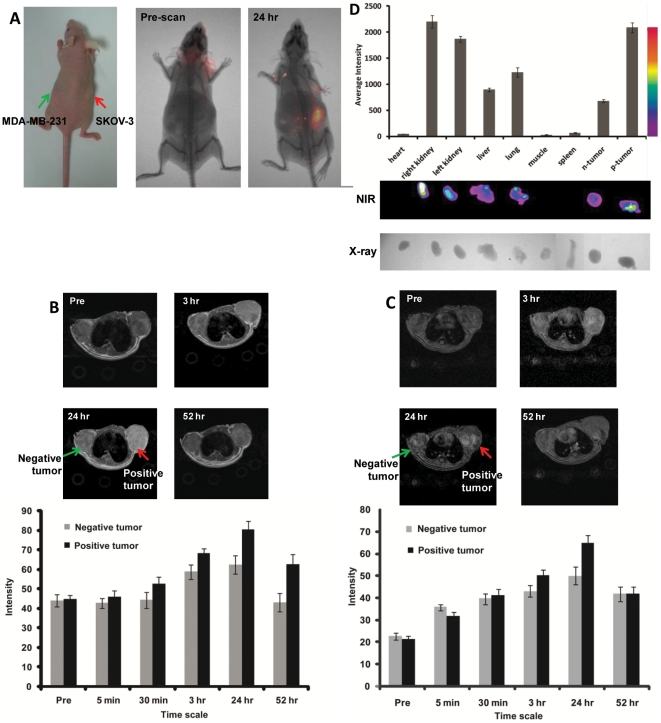
In vivo cancer imaging of contrast agent uptake, retention and distribution in multiple different normal host organs and in both SKOV-3 (HER2 positive, right) and MDA-MB-231 (HER2 negative, left) xenografts in balb/c nude mice (Charles River laboratory). (**A**) NIR fluorescence imaging (Kodak 8000) revealed that ProCA1-Affi is able to target to the HER2 positive tumor (SKOV-3, right) 24 hr after injection from tail vein. No significant near IR signal was detected in the HER2 negative tumor (MDA-MB-231, left) (n = 6, P<0.05). (**B**) Fast spin echo and (**C**) gradient echo transversal MR images collected prior to injection and at various time points post injection of 3.0 mM of ProCA1-affi-m in HEPES saline via tail vein. The MRI signal on the positive tumor (SKOV-3, right) exhibits significant enhancement at 3 hr post injection and reaches maximum enhancement at 24 hours post injection (n = 6, P<0.05). The slight differences in MRI signals result from the use of different pulse sequences for imaging (Methods). (**D**). NIR images of the dissected mouse organs. General bio-distribution was obtained based on the NIR signal and western blot assay. The ProCA1-affi-m mainly distributed in the positive tumor, liver and kidney.

We then tested whether our designed contrast agent would result in MRI contrast enhancement in xenograft models of these two human cancer cell lines. Due to less efficiency in formation of xenograft tumors in nude mice using the AU565 cell line, and extremely fast growth rate of the mouse breast cancer cell line EMT-6 in xenograft, we switched to a very commonly used ovarian cancer cell line SKOV-3 with a high HER2 expression and a breast cancer cell line MDA-MB-231 with low expression level of HER2. Xenograft models of these two cell lines had very similar growth rates in nude mice. The SKOV-3 tumor cells were subcutaneously implanted in the right flank, while the MBD-MDA-231 with a low HER2 expression was implanted in the left flank of the same mouse for direct comparison (Fig. 3A). The contrast agent Gd^3+^ ProCA1-affi-m at concentration of 3 mM (100 fold lower than clinically-approved contrast agent DTPA) was administrated via the tail vein (80 µl of each mouse, n = 6). Pre- and post-contrast MRI were collected at different time points using T1 and T2 weighted fast spin echo or T1 weighted gradient echo sequences. At 3 hour time point, HER2 positive tumor exhibited significant contrast enhancement. Strong contrast enhancement was observed in the SKOV-3 tumor 24 hours after injection, while there were much less changes in contrast in the MBD-MDA-231 tumor (Fig. 3B, C). Such MRI contrast enhancement was decreased after 24 hrs post injection. In parallel, the mice were imaged using an optical animal imaging system (Fig. 3A). Consistent with MR imaging, we observed a strong NIR light emission from the SKOV-3 tumor at 24-hour post-administration of the contrast agent, however, the NIR intensities at the MBD-MDA-231 tumor site were much less than that of the SKOV-3 tumor (Fig. 3B).

### Histology analysis of distribution and permeability

To further analyze the HER2 targeting properties of the protein contrast agent, tumors and organs from the imaged mice were collected 48 hours after administration of the agent (Fig. 3D). The organs and tumors were imaged using optical animal imaging. It was clear that there were very high levels of accumulation of Cy5.5 in the liver, kidneys, and the SKOV-3 tumor. There were medium levels of the NIR dye at lung. In comparison, the level of Cy5.5 at the MBD-MDA-231 tumor was quite low (Fig. 3D). The results strongly suggested that our protein contrast agent led to the HER2 specific MR image enhancement.

**Figure 4:**
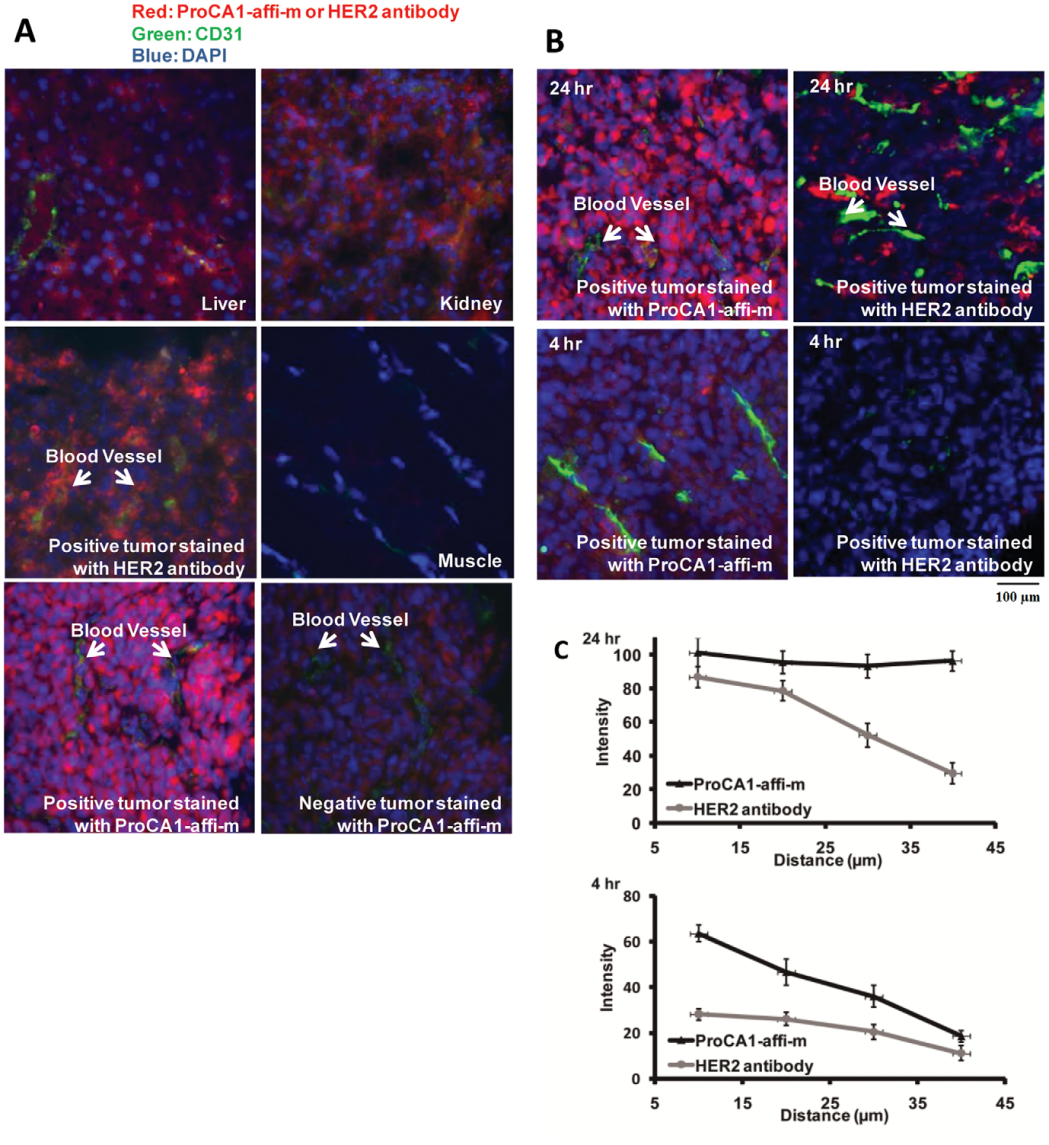
Histological examination of contrast agent uptake, retention and distribution in multiple different normal host organs and in both SKOV-3 (HER2 positive, right) and MDA-MB-231 (HER2 negative, left) xenografts in balb/c nude mice (Charles River laboratory) (n = 6, P<0.05). (**A**) Immune histology fluorescent (IHF) staining was applied to various tissue slides stained by antibody against ProCA1-affi-m (red), Blood vessels biomarker CD31 (green), and nucleus DAPI (blue). The slides stained without primary antibody were used as blank control. (**B, C**) The tissue penetration properties of ProCA1 –affi-m were compared with antibody by IHF staining. The tumor slides are from the mice which were dissected 24 hr and 4 hr after injection with ProCA1-affi-m or antibody. After 4 hr, ProCA1 –affi-m began to distribute around the blood vessel. The antibody had not been detected in the tumor tissue. After 24 hr, ProCA1-CD2-affi was evenly distributed in the tumor tissue and the antibody mainly concentrated around the blood vessel. The scale bar value is 100 µm.

To further verify the contrast agent targeted to the HER2 positive tumor, we carried out immunohistochemistry (IHC) staining using the antibody PAbPGCA1 with tissue slides made from the tumor samples collected from the imaged mice as well as selected organs. The strongest staining was observed with liver and the SKOV-3 tumor tissue slides (Fig. 4A). Close examination of the staining patterns of the tumor slides revealed distribution of the designed protein both inside and outside the cancer cells with substantial stronger staining inside the cancer cells, indicating internalization of the protein contrast agent. This staining pattern provided a strong support for the cancer cell targeting by the contrast agent. The kidney slides also gave strong immunostaining consistent with the NIR imaging finding. Interestingly, the areas near proximal tubes showed the strongest staining (Fig. 4A), suggesting that the protein contrast agent may be secreted through the kidney. This is consistent with observations that there were good levels of both Gd^3+^ (by γ-counting of ^153^Gd^3+^) and the protein (by NIR fluorescence) in the urine of mice that were injected with the contrast agent (data not shown). Immunostaining of tissue sections from MBD-MDA-231 tumor revealed very weak staining (Fig. 4A).

To further verify the HER2 specific MRI contrast enhancement, we carried out a competition assay based on the assumption that if our protein contrast agent targeted HER2 and led to HER2 specific MRI contrast enhancement, affibody alone would be a strong competitor for the binding to the cell surface HER2 and consequently block the binding by our designed protein. Nude mice that carried SKOV-3 tumors were pre-injected with buffer saline or 3 mM of HER2 affibody Z_HER2-342_ labeled with Cy5.5 twice at 12 hr and 2 hr. Gd^3+^ ProCA1-affi-m (80 µl) at a concentration of 3 mM was subsequently administrated to the mice by intravenous injection. The mice were then scanned at a 4.7 T MRI scanner via the same procedures. Our results demonstrated that the MRI contrast enhancements were not observed at the SKOV-3 tumor site in the mice that received HER2 affibody labeled with Cy5.5, while the contrast enhancements in the liver and kidney in the same mouse were not affected by the administration of HER2 affibody (Supporting File S1). NIR imaging did exhibit high intensity in the tumor, which indicates that the affibody binds to the positive tumor and blocks the binding of MRI contrast agents. Conversely, the administration of the saline prior to injection of the designed protein contrast agent did not block the MRI contrast enhancement (Supporting File S1). The results with HER2 affibody blocking strongly support our conclusion that the MRI contrast enhancement from administration of Gd^3+^ ProCA1-affi-m is HER2 specific.

A very crucial requirement for application of an agent for delivery of both drugs and imaging probes to target a disease marker is the capability of the agent to cross the endothelial barrier and to allow for proper tissue penetration and distribution. In particular, even distribution of an imaging probe throughout the entire cancer site is vitally important for quantitative or semi-quantitative assessment of a particular cancer marker. HER2 is evenly expressed across the entire SKOV-3 tumor as revealed by immunostaining using a commercially available antibody (Sigma). Co-staining using an antibody against the endothelial marker CD31 revealed that the distribution of HER2 is not dependent on the distance to the vessels (Supporting File S1). Presumably, the proper size of ProCA1-affi-m provides a great advantage to target the molecular markers. To evaluate the tissue distribution and endothelial penetration of our designed protein contrast agent, we conducted immunofluorescence staining of the designed protein in the tissue sections prepared from various organs after systematic administration of the protein using the antibody PAbPGCA1. The tissue sections were also co-stained with the antibody against CD31. It was clear that high levels of ProCA1-affi-m were targeted to the SKOV-3 tumor at 24 hours post injection, and the protein was distributed in the entire tumor evenly since its intensity is not changed significantly upon increasing the distance from vessel staining CD31 to 40 µm (Fig. 4B). The results from the immunofluorescence staining suggested that the designed protein contrast agent had excellent endothelial and tumor tissue penetration, and was not simply trapped in the blood in the micro-vasculature of the tumor tissue.

Since antibodies have been widely used in drug and imaging probe deliveries in molecular marker targeted applications [4], [6] we further compared the immunofluorescence staining patterns of our designed protein agent and a commercially available HER2 antibody. To this end, ProCA1-affi-m (10 mg/kg) or the HER2 antibody (10 mg/kg) was administrated in the SKOV-3 tumor bearing nude mice via tail vein. 24 hours post injection, tissue sections were prepared from the tumor tissue, and the sections were analyzed either by immunofluorescence staining using the antibody PAbPGCA1 (for analyses of ProCA1-affi) or direct application of the second antibody against rabbit IgG to detect the bound anti-HER2 antibody. The tissue sections from both cases were also co-stained with the antibody against CD31. At 24 hours post injection, the anti-HER2 antibody was mainly concentrated around endothelial cells as revealed by co-localization with anti-CD31 staining. This is in sharp contrast to the even distribution of ProCA1-affi-m in the entire tumor (Fig. 4B, C). The distributions of the anti-HER2 antibody to the area distant from endothelial cells were clearly quite reduced as demonstrated by weak immunostaining in the areas where there was no CD31 staining (Fig. 4B, C). We further examined the distribution of our protein agent and the anti-HER2 antibody at an early time point. Tissue sections from the SKOV-3 tumors were prepared 4 hours after administration of ProCA1-affi-m or the anti-HER2 antibody. Interestingly, while the ProCA1-affi-m was largely concentrated with the CD31 staining in the tumor, the anti-HER2 antibody was not detectable by the immunofluorescence staining analyses. (Fig. 4C). The results strongly suggested that our designed protein agent was able to cross the endothelial and distribute to the deep tumor tissue a few hours after administration while the large size of antibody (∼160 kDa) significantly hindered endothelial and tissue penetration. Consistent with the 50% reduction of MRI intensity at the tumor site by affibody blocking shown in Supporting File S1, the fluorescence immunostaining at the same tumor site also exhibited about 60–90±20% decrease in intensity (n = 6, P<0.05) (Fig. 4C). Taken together, our developed MRI contrast agent exhibits a potential capability for future quantitative analysis of the biomarker *in vivo*.

HER2 has been validated as a very important prognosis and treatment marker for cancer patients expressing HER2, especially in the case of breast cancer. Development of Herceptin (trastuzumab) and other HER2 targeting drugs has resulted in significant improvement in patient survival. Unfortunately, current methods for determination of HER2 status rely on invasive biopsy coupled with IHC using a qualitative scoring system [15]. These methods suffer from both high false positive and false negative results, and large discordance in detection of HER2 expression in primary tumors and metastases due to heterogeneity in tissue sampling. These methods also cannot detect HER2 expression levels and patterns in the entire cancer site. According to a recent study by Philips *et al*., one in five HER2 clinical tests provided incorrect results [16]. Therefore, there is a great need to develop MRI contrast agents with specificity and sensitivity for HER2 imaging [17].

## Discussion

In this present study, we demonstrate the success in molecular imaging of HER2 by developing a novel class of multiple modality contrast agent. To our knowledge, there is no previous report of effective imaging of HER2 expression cancer *in vivo* by noninvasive MRI with desirable tissue penetration and using only a single injection. Our approach in designing protein-based molecular imaging contrast agent differs greatly from previous reported studies in several aspects and represents a significant advance in molecular imaging by MRI. First, high relaxivity value in both T1 and T2 achieved by designing a Gd^3+^ binding site into a stable scaffold protein [18] allows for increased sensitivity in the detection of disease markers by MRI. Our achievement of MR imaging in animal with 100-fold lower dose usage than clinically used non-targeting agent DTPA is also likely due to improved pharmokinetic properties such as retention time and biodistribution. Such significant improvements in *in vivo* dosage efficiency will potentially reduce potential Gd^3+^ toxicity risks, such as NSF (Nephrogenic Systemic Fibrosis). Second, the relatively small molecular size of the designed agent provides a unique opportunity to target the imaging probe to the molecular marker in the entire tumor mass. This property is of vital importance, especially for quantitative assessment of the molecular marker based on the imaging results. Several approaches have been employed to develop targeted MRI contrast agents [5], [6], [19], [20], [21], [22], [23], [24], [25]. To increase contrast effects, high pay load contrast molecules were created by either encapsulating a large number of Gd-DTPA, conjugating multiple contrast agents such as polylysine-Gd-DTPA (PAMAM) [26], or using supermagnetic iron oxide nanoparticles [27], [28]. The antibody approach was widely utilized as the targeting moiety either directly conjugated with high payload contrast agent or elegantly applied in multiple steps to pre-label the tumor as a biotin-labeled antibody [29]. These pioneering studies demonstrated the feasibility of the targeting approach; however, the large size of the antibody-conjugated imaging probes is likely to severely limit the endothelial penetration and even-distribution of the probes in the whole tumor (Fig. 4B and C). On the other hand, our contrast agent exhibits endothelial penetration capabilities and an excellent distribution in the entire cancer mass as revealed by its adequate distribution near the blood vessel four hour after administration, and the nearly-uniform distribution observed 24 hours post injection. One potential application of our developed MRI contrast agent is for quantitatively or semi-quantitatively assessing the HER2 levels in the entire tumor site using MR imaging, which is impossible with any current methods. Since HER2 is overexpressed in a large percentage of breast, ovarian, gastric, urinary bladder and a number of other carcinomas, the developed contrast agents may be beneficial for imaging of HER2 in several types of cancer. *In vivo* real time monitoring of the changes in HER2 expression levels and patterns will provide vital information for evaluation of the efficacy of drug treatments and for designing further strategies for cancer treatments.

## Materials and Methods

### Ethics Statement

All the mice in this research were inoculated with human cancer cell lines (SKOV-3 and MDA-MB-231) subcutaneously. All the contrast agents were injected from tail vein. The animal research has been proved by IACUC (Institutional Animal Care and Use Committee) of Georgia State University. The permit number of our protocol is A06007. All the cancer cell lines used are commercial available from ATCC.

### Relaxivity and metal binding affinity measurement

Relaxation times, T1 and T2, were determined on the 1.41T Minispec Relaxometer (mq60 NMR Analyzer, Bruker) at 37°C. The ProCA1-affi and ProCA1-affi-m (modified by PEG) were diluted with 10 mM Tris buffer, pH 7.0. Proteins prepared with a series of concentrations: 40–120 µM, were applied for the relaxation time measurement. The relaxivities, r_1_ and r_2_, were obtained by fitting the relaxation times as a function of the Gd^3+^ concentrations (Fig. 1b). The Gd^3+^ -binding affinities with ProCA1-affi and ProCA1-affi-m were investigated by the competitive assay with the dye Fluo5N (a metal ion indicator, Invitrogen Molecular Probes). The fluorescence spectra were collected on a fluorescence spectrophotometer (Photon TechnologyInternational, Inc.) with a 10 mm path length quartz cell at room temperature [10].

### Tumor cell targeting

The AU565 (ATCC), originally from human breast cancer, has an expression level of HER2 at about 10^6^ per cell. The EMT6 (ATCC) is a HER2 negative cell line from mouse breast cancer. The ProCA1-affi and ProCA1-affi-m were incubated with the two kinds of cells at 4 and 37°C, respectively, for 1 hr. Then the cells were washed 3 times, 5 min each with Tris buffer. The primary antibody was generated on rabbit by using ProCA1-affi-m as antigen. The secondary antibody was FITC conjugated (Invitrogen). Finally, the cells were mounted with Prolong antifade reagent (Invitrogen). In the ELISA assay, the secondary antibody was HRP conjugated and reacted with OPD for 5 min, and optical density was measured at 490 nm. In the radioactive assay, the ProCA1-affi binding with ^153^Gd^3+^ was used to treat the cancer cells; the radioactivity of the cell pellets was measured by γ counter after washing 5 times.

### Animal Model

The Balb/c nude mice were injected with ∼2×10^6^ SKOV-3 (ATCC) and MDA-MB-231 (ATCC) cells (in 100 µl matrix gel and saline mixture) subcutaneously on the right and left back respectively. The xenografts were established during 4–6 weeks until the tumor diameter reached approximately 5 to 10 mm.

### *In vivo* imaging

The ProCA1-affi-m injected into the xenografts was concentrated in ∼5 mM in HEPES buffer, pH 7.0. The 100 µL of ProCA1-affi-m was injected to each xenograft by i.v. injection. The MR images were taken at various time points: 30 min, 4 hr, 24 hr and 48 hr using a 4.7 T scanner. The NIR images were taken at 4 hr, 24 hr and 48 hr.

The mice were imaged using two pulse sequences: the T1 and T2 weighted fast spin echo sequence (TR = 2 s, TE = 0.022 or 0.066 s) and the T1 weighted gradient echo sequence (TR = 0.088 s, TE = 2 s and P = 0.009 s). The fields of view are 3 cm×3 cm with matrix of 256×256 and slice of 1 mm in thickness. Image J was used to quantitatively analyze the MRI images obtained. The regions of interest (ROI) were selected by circling the tumor sites. Then the signal intensities of the ROIs were calculated and compared. Six adjacent slides were selected to measure signal changes which were averaged to obtain statistical significant results.

### Histology analysis

The mice were sacrificed after taking final images. Primary organs, such as livers, kidneys, lungs, spleens, muscle and tumors were dissected out for histology analysis. The tissues were frozen in liquid nitrogen immediately following dissection. Then the frozen tissues were sliced into µm thin sections. The sections were triple stained with antibody against ProCA1-affi-m (red), CD31 antibody (green) and DAPI (blue). Quantitative analysis on the tissue slides was measured by the software Image J. Statistical results were obtained from 2 tumor slides, and 3 view regions were taken from each slide. Detection of the antibody decreased with increasing distance from the blood vessel. The ProCA1Affi-m was found to be well distributed throughout the whole tumor.

## Supporting Information

File S1 **(A)** Magnetic resonance images and image intensities of the mouse tumor pre-blocked by affibody Z_HER2:342_ **(B)** Purification of PEGylated ProCA1-affi-m **(C)** Far-UV CD and Tryptophan fluorescent spectra **(D)** Optical spectrum of ProCA1-affi-m conjugated with NIR dye Cy5.5 **(E)** NIR images of cultured cancer cells (AU565 and SKOV-3) with high expression of HER2. The scale bar value is 25 µm. **(F)** Immunofluorescent histology of tumor tissue (Xenograft SKOV-3 model) stained by HER2 antibody and ProCA1-affi-m. The scale bar value is 100 µm. **(G)** Blood circulation of GdCl3, ProCA1-affi and ProCA1-affi-m in Xenograft nude mice **(H)** Toxicity analysis by clinical chemistry assay.
(DOC)
