# Supplementary material for: HER2 Targeted Molecular MR Imaging Using a De Novo Designed Protein Contrast Agent
Source: PLoS One. 2011 Mar 24;6(3):e18103. doi: 10.1371/journal.pone.0018103 (PMC3063795; doi:10.1371/journal.pone.0018103)
Supplement: File S1 — (A) Magnetic resonance images and image intensities of the mouse tumor pre-blocked by affibody ZHER2:342 (B) Purification of PEGylated ProCA1-affi-m (C) Far-UV CD and Tryptophan fluorescent spectra (D) Optical spectrum of ProCA1-affi-m conjugated with NIR dye Cy5.5 (E) NIR images of cultured cancer cells (AU565 and SKOV-3) with high expression of HER2. The scale bar value is 25 µm. (F) Immunofluorescent histology of tumor tissue (Xenograft SKOV-3 model) stained by HER2 antibody and ProCA1-affi-m. The scale bar value is 100 µm. (G) Blood circulation of GdCl3, ProCA1-affi and ProCA1-affi-m in Xenograft nude mice (H) Toxicity analysis by clinical chemistry assay. (DOC) [file pone.0018103.s001.doc]

Supporting information online for

**HER2 Targeted Molecular MR Imaging Using a *de novo* Designed Protein Contrast Agent**

Jingjuan Qiao, Shunyi Li, Lixia Wei, Jie Jiang, Robert Long, Hui Mao, Wei Ling, Liya Wang, HuaYang, [Hans E.Grossniklaus,](https://webaccess.gsu.edu/gw/webacc)  and Zhi-Ren Liu, Jenny J. Yang

1. **Molecular cloning and protein generation**

A Gd3+ binding site was designed to the original scaffold protein ProCA1-CD2. The affibody Z_HER2:342_ was cloned to the C-terminal of ProCA1-CD2 with a GGSGG linker in pGEX-2T vector (Fig. 1A). The fusion protein ProCA1-affi was purified by GS-4B column and PEGylated by P40 reagents[1]. Finally the protein was further purified by cation exchange column (Fig. S2). The PEGylated protein ProCA1-affi-m was further conjugated withCy5.5 by the Cysteine at the C-terminal of ProCA1-affi-m.

1. **Blocking assay with affibody Z_HER2-342_**

In the blocking experiment, the mice were born with SKOV-3 tumor on the right back. The affibody ZHER342 of 3 mM in HEPES buffer was intravenous injected into the mice for 12 hr and 4 hr before taking MR images[2]. The mice were also scanned at various time points of 4 hr and 24 hr.

1. **Protein Purification and Modification**

In order to increase the stability and solubility of ProCA1-affi, the fusion protein was modified by PEGylation with PEG40. The activated PEG reagent was mixed with ProCA1-affi in 5:1 ration in phosphate saline, pH7.0. After 1 hr reaction, the reaction was ended by adding free amino acids. Then the PEGylated ProCA1-affi-m was purified by SP cation exchange column (Fig. S2). After purification, the free PEG reagent has been removed. The SDS-PAGE gel has been stained with iodine and commas blue in consequence (Fig. S2). The gel also shows ProCA1-affi has been PEGylated with various numbers of PEG units.

1. **Examination of protein folding**

The tryptophan fluorescent spectrum and far UV CD were used to exam the folding of ProCA1-affi. The excitation wavelength for the fluorescent scan is 280 nm; the emission range is 260-420 nm. The far UV CD spectrum at the range of 190-260 nm was scanned to compare the secondary structure of ProCA1-affi with its original protein ProCA1.CD2.

1. **Conjugation of NIR dye Cy5.5 to ProCA1-affi-m**

The ProCA1-affi was conjugated with the NIR dye Cy5.5 at the C-terminal of the Cys residue. The UV absorbance spectrum indicates that 20% of the ProCA1-affi has been conjugated with the NIR dye.

1. **Comparison of Tissue Penetration between HER2 antibody and ProCA1-affi-m**

The mice were injected with ProCA1-affi and HER2 antibody respectively in the dosage of 200 µM in 100 µl saline buffer. After 4 hr and 24 hr, different mice are sacrificed and the tumor tissues were collected for histology analysis. The tumor slides from the control mice were also stained directly by ProCA1-affi and antibody. The Fig. S6 shows that the receptors of HER2 are evenly distributed around the blood vessel (green).

1. **Blood Circulation of ProCA1-affi-m**

ProCA1-affi binding with ^153^Gd^3+^ was injected into the xenograft mice for blood circulation analysis. The blood was collected at various time points: 1 hr, 4 hr, 8 hr and 12 hr [3]. Fig. S7 shows that the PEGylated ProCA1-affi-m has much longer circulation time comparing with the non-PEGylated ProCA1-affi-m. In the control group, GdCl_3_ had been circulated out in 1 hr.

1. **Toxicity of ProCA1-affi-m**

The 2 mM of ProCA1-affi-m in 100 µl saline was injected into the regular CD1 mice for toxicity analysis. The control group was injected with 100 µl saline only. After 2 days, the mice were sacrificed and blood was collected to get the blood serum. The enzymes of creatinine and ALT, ALP in the blood serum were measured to analyze the toxicity in kidneys and liver (Research Animal Diagnostic Laboratory, University of Missouri). Comparing with the control group, the Supplementary Table 1 indicates our ProCA1-affi-m has relatively low toxicity.

1. Magnetic resonance images and image intensities of the mouse tumor pre-blocked by affibody Z_HER2:342_

**(B)** Purification of PEGylated ProCA1-affi-m

**(C)** Far-UV CD and Tryptophan fluorescent spectra

**(D)** Optical spectrum of ProCA1-affi-m conjugated with NIR dye Cy5.5


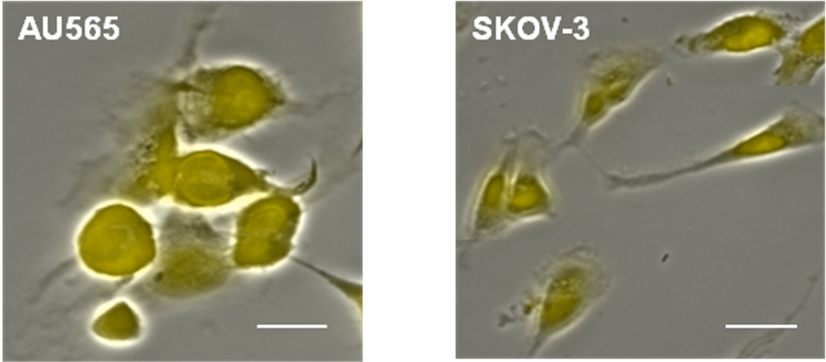


**(E)** NIR images of cultured cancer cells (AU565 and SKOV-3) with high expression of HER2. The scale bar value is 25 µm.


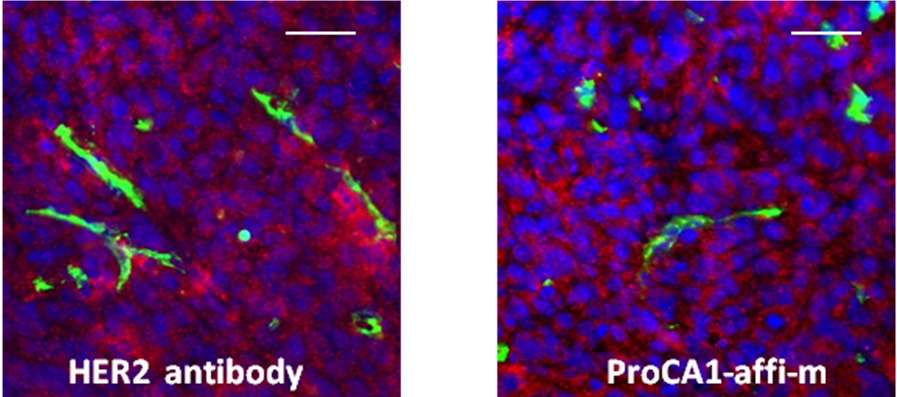


**(F)** Immunofluorescent histology of tumor tissue (Xenograft SKOV-3 model) stained by HER2 antibody and ProCA1-affi-m. The scale bar value is 100 µm.

**(G)** Blood circulation of GdCl_3_, ProCA1-affi and ProCA1-affi-m in Xenograft nude mice

**(H)** Toxicity analysis by clinical chemistry assay

**References:**

1. Akiyama Y, Mori T, Katayama Y, Niidome T (2009) The effects of PEG grafting level and injection dose on gold nanorod biodistribution in the tumor-bearing mice. J Control Release 139: 81-84.

2. Banerjee SR, Foss CA, Castanares M, Mease RC, Byun Y, et al. (2008) Synthesis and evaluation of technetium-99m- and rhenium-labeled inhibitors of the prostate-specific membrane antigen (PSMA). J Med Chem 51: 4504-4517.

3. Kramer-Marek G, Kiesewetter DO, Martiniova L, Jagoda E, Lee SB, et al. (2008) [18F]FBEM-Z(HER2:342)-Affibody molecule-a new molecular tracer for in vivo monitoring of HER2 expression by positron emission tomography. Eur J Nucl Med Mol Imaging 35: 1008-1018.
